# Electro-chromic structure with a high degree of dielectric tunability

**DOI:** 10.1038/s41598-019-47233-1

**Published:** 2019-07-24

**Authors:** S. Bulja, R. Kopf, A. Tate, T. Hu, R. Cahill, M. Norooziarab, D. Kozlov, P. Rulikowski, W. Templ

**Affiliations:** 1 Bell Labs Ireland, Blanchardstown Industrial Park, Dublin 15, Ireland; 2 Bell Labs, 600 Mountain Ave., Murray Hill, NJ 07974 USA; 3 Queen’s University Belfast, School of Electronics, Electrical Engineering and Computer Science, BT3 9DT Belfast, United Kingdom; 4 Trinity College Dublin, School of Physics, 42 Pearse Street, Dublin 2, Ireland; 5 Bell Labs Germany, Lorenzstrasse, 10, BW, 70435 Stuttgart, Germany

**Keywords:** Electrical and electronic engineering, Electronic devices, Electronic and spintronic devices

## Abstract

In conjunction with their electronically reconfigurable optical properties, inorganic, WO_3_/LiNbO_3_/NiO Electro-Chromic materials (EC) have recently been shown to exhibit a degree of electric field induced dielectric tunability at radio frequencies, to the level comparable with more mature bulk-tuneable technologies. However, the full extent of their dielectric tunability remains fully unexplored, due to a fundamental lack of understanding of its intricate tuning mechanisms. The unveiling of their tuning principles is paramount towards a comprehension of not only their optical and radio frequency dielectric tunability, but also for the creation of EC structures with substantial permittivity tuning ratios. Here, we report on an inorganic, WO_3_ and LiNbO_3_ – based EC structure with perturbed constituent layers. We developed and synthesised a new EC structure by inserting the chromic layers in the interior of the device and partitioning the electrolyte layer and assigning it to the device’s peripheries. This new arrangement allows for an increase in the dielectric tunability of over three times compared to previously reported standard EC structures in the frequency range from 1–20 GHz.

## Introduction

**Figure 1:**
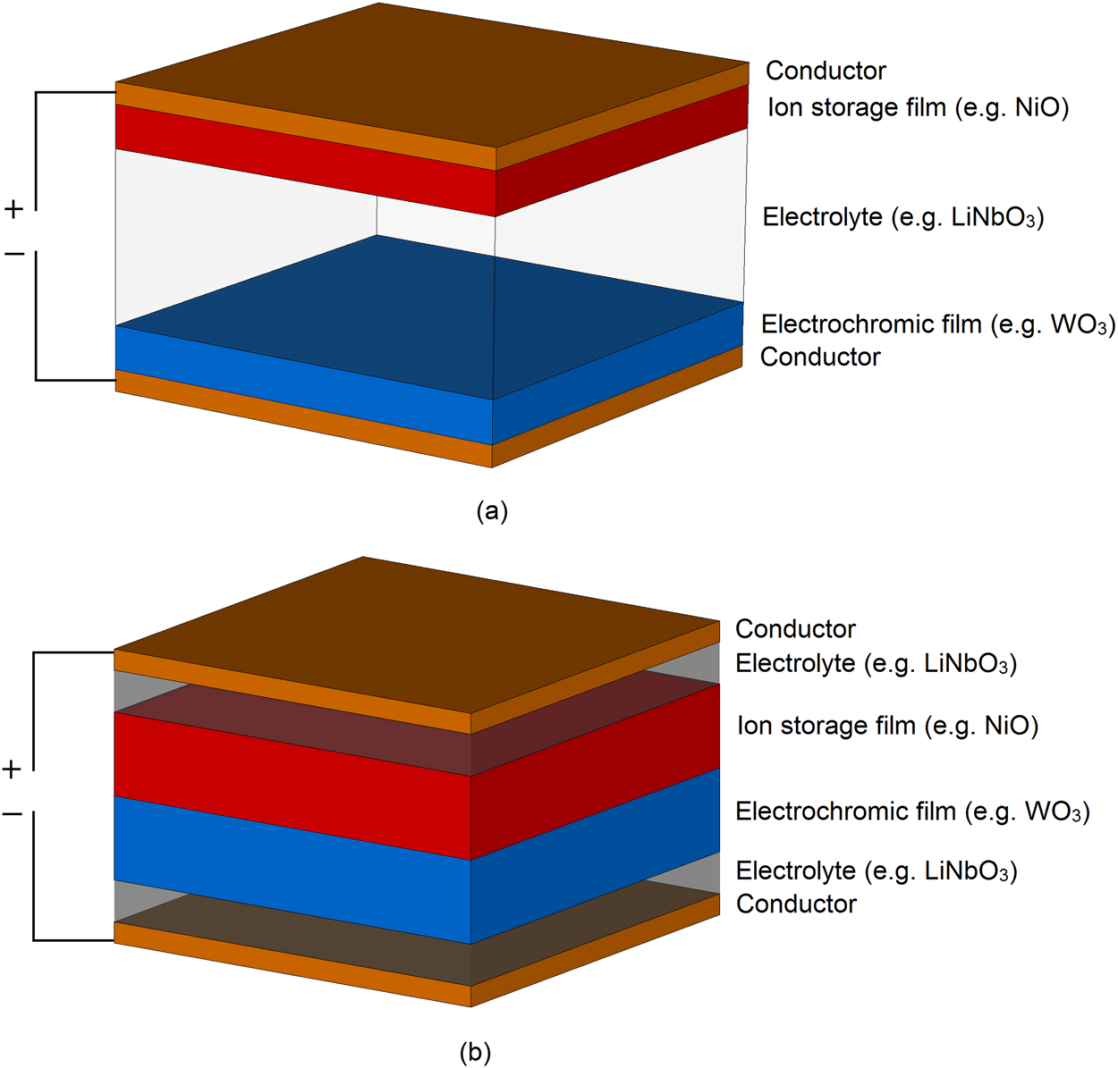
Structures of inorganic EC cells: (**a**) standard. (**b**) proposed.

Electro-chromic (EC) materials is an emergent class of electrically bulk-tuneable dielectrics. Their dc-bias induced optical tunability, discovered by Deb^1^, was the subject of extensive research^2–18^, however, research on their bulk, microwave and millimetre wave tuneable dielectric characteristics is still in infancy. With the exception of recent work^19–21^, which showed that inorganic EC materials not only possess frequency dependent dielectric tunability, but also demonstrated that the absolute and relative values of dielectric tunability can be tailored, there is very limited information available in the open literature on their dielectric characteristics. It was reported^20^ that the variation of the height of the LiNbO_3_ layer in a standard, complimentary WO_3_/LiNbO_3_/NiO EC stack, from 500 nm to 700 nm, results in a change of its relative dielectric tuneable range from 17% to 20% and in a change of its minimum relative dielectric permittivity from 24 to 28, at 1 GHz, respectively. The dielectric tuneable range of EC material is on par with some other state-of-the-art bulk tuneable technologies, such as Liquid Crystals^22–26^ (LC). Even though it may be pertinent to speculate that the extent of dielectric tunability of inorganic EC materials can be increased using a different chemical composition of the constituent materials, there are certain elementary limitations, which impede the degree of their tunability. It is, however, consensually agreed that the origin of EC tunability stems from the redox processes taking place inside the EC cell, which, ultimately, result in the Mott transition^27^ of the transition-metal oxide (chromic) layers, Fig. 1a.

With reference to Fig. 1a, the total equivalent capacitance of the EC structure is the sum of the capacitances of the constituent layers, WO_3_, LiNbO_3_ and NiO. In turn, these capacitances are directly proportional to their corresponding dielectric permittivities, by $C=\varepsilon \frac{A}{d}$, where A and d refer to the electrodes’ surface area and separation, respectively. By EC cell actuation, the redox processes are activated and ions and electrons migrate outside of the electrolyte layer and get injected into the chromic (WO_3_ and NiO) layers. The migration of the charges from the electrolyte layer results in the reduction of its equivalent capacitance. These expelled ions and electrons are subsequently injected into the chromic, transition metal-oxide layers, located at the bottom and top electrodes, initiating their transition from insulators to conductors. Given their electrically small thicknesses at micro- and millimetre wave frequencies, this effect manifests itself not only as an increase of their equivalent capacitance, but it also results in an effective reduction of the height of the composite, non-conductive EC structure, i.e. h_EC_ = h_WO3_ + h_LiNbO3_ + h_NiO_ ~ h_LiNbO3_, since the chromic layers in this case become, effectively, part of the top and bottom electrodes, respectively. At micro- and millimetre wave frequencies, the observable net effect of channel shortening manifests itself in the increased loss tangent and reduced dielectric tunability in the actuated state compared to the non-actuated state. At optical wavelengths, this phenomenon is perceived as the darkening of the EC cell.

**Figure 2:**
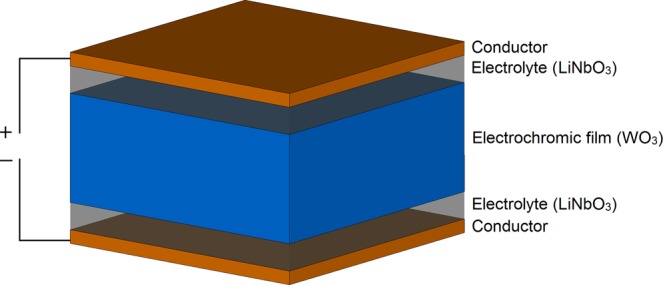
Structure of manufactured EC cell.

Here, we report on an inorganic, WO_3_ and LiNbO_3_ – based EC structure with perturbed constituent layers, envisioned to address the channel shortening effect, Fig. 1b, without affecting the EC cell’s optical characteristics. We developed and synthesised the EC structure by allotting the chromic layers (Electro-chromic and Ion Storage films) to the interior of the device and partitioning the electrolyte layer and assigning it to the device’s peripheries. This arrangement prevents the shortening effect of the structure upon the dielectric-conductor transition, due to the shielding nature of the electrolyte layer, which is in direct contact with external electrodes, while maintaining optical darkening. In essence, the effective non-conductive height of the channel of our EC structure remains the same in the non-actuated and actuated states. The power of this EC assembly is ultimately demonstrated in a significantly greater dielectric tunability than previously reported with standard EC structures, while still being producible using standard micro-fabrication techniques. In the scenario depicted in Fig. 1b, the chromic films are in direct contact with each other, however, just like in the case of a standard EC structure of Fig. 1a, the Ion Storage film is not necessarily required for the correct operation of the proposed EC cell. The EC cell reported in this paper does not feature the Ion storage (NiO) layer – instead it consists only of the WO_3_ active layer, Fig. 2.

## Results

**Figure 3:**
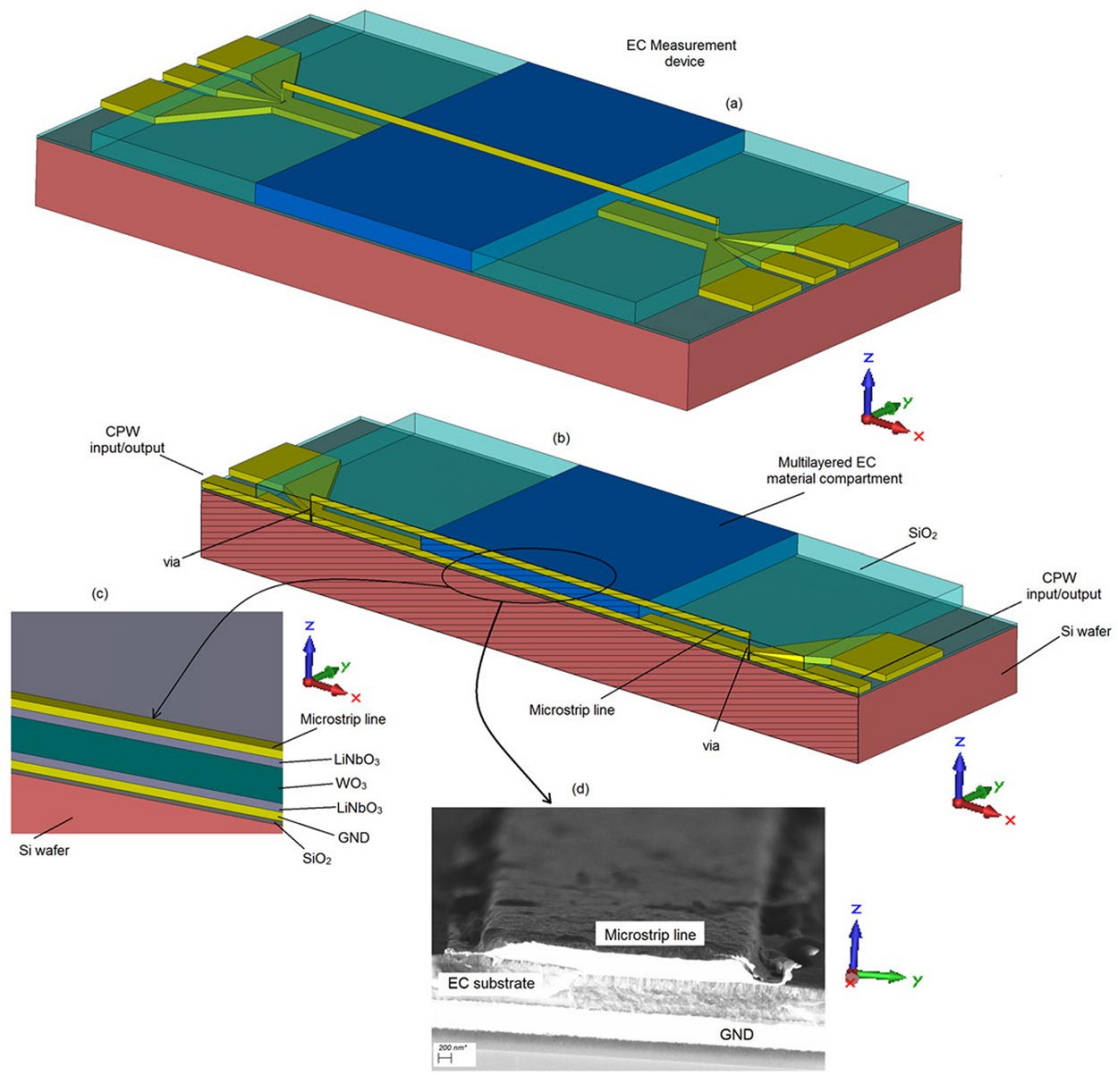
EC based measurement device. (**a**) Entire test cell. (**b**) Cross section view. (**c**) Enlarged side view of the EC section. (**d**) cleaved image of the EC cell, showing the top gold line, EC substrate and the ground plane obtained using Field Emission Scanning Electron Microscopy (FESEM).

The measurement of the RF characteristics of the proposed EC structure is performed using a test cell arrangement previously developed^20^, Fig. 3a. The device was, however, modified so that it can house the proposed EC cell. Its structure consists of the EC cell under test and two broad-band Coplanar Wave Guide (CPW) to a microstrip line transitions connected at each end of the EC cell.

The device fabrication has been described previously^19,20^ and the structure is shown in Fig. 3(a–c) (not to scale). The substrate is 600 µm, n-doped Si, with a surface resistivity of 10 Ω.cm and dielectric constant of ε_rSi_ = 11.9. A thin dielectric layer of SiO_2_ with a height of h_trench_ = 300 nm is deposited on the Si to separate the conductive Si layer from the gold ground plane. The SiO_2_ layer was deposited using Plasma-Enhanced Chemical-Vapor-Deposition. It was then lithographically patterned and plasma etched to expose the contact pads and open the vias. The thickness of the gold ground plane, containing the patterned CPW, is h_b,cond_ = 500 nm. The width of the top Au electrode and, hence, the anode of the EC cell is W_EC_ = 5 µm and its length is L_EC_ = 2 mm. The top electrode is deposited partially on the SiO_2_ substrate and partially on the composite EC material. The EC layers, WO_3_ and LiNbO_3_, were patterned using a standard resist and lift-off technique. The thicknesses of the bottom and top LiNbO_3_ layers are the same and correspond to h_LiNbO3_ = 150 nm, whereas the WO_3_ layer thickness is h_WO3_ = 140 nm, rendering the height of the proposed EC cell to h_EC_ = h_LiNbO3_ + h_WO3_ + h_LiNbO3_ = 440 nm. All of the individual layers were characterized previously using Stylus profilometry. In addition, ellipsometry and interferometry were used to determine the refractive indices and the thicknesses of the individual WO_3_ and LiNbO_3_ layers. Figure 3d shows an image of a cross-section of the finished device made by the Field Emission Scanning Electron Microscopy (FESEM, Carl Zeiss Ultra) method using a secondary electron detector at an accelerating voltage of 4 kV. The measurements of the scattering (S)-parameters of the proposed cell were performed using a Rohde-Schwarz Vector Network Analyser from 1 GHz to 20 GHz, with an average power provided at the input of the measurement device of 5 dBm. The EC cell is dc-biased through a low-pass filter connected in parallel with one of the device’s terminals. Since the EC cell is electrically thin, it is desirable to have the knowledge of its dielectric breakdown voltage, so that the dc actuation voltage does not exceed it. However, the maximum dc voltage (dielectric breakdown voltage) that can be applied to any dielectric is strongly dependent on its dielectric characteristics (permittivity and loss). Since in the present case, the dielectric characteristics of the EC cell are sought, and, therefore, not known *a priori*, it is not possible to theoretically determine the dielectric breakdown voltage, which would establish the upper, safe dc-voltage limit. Given this limitation, the EC cell in this work is gradually activated by applying the dc bias voltage in sufficiently small increments and allowing adequate settling time at each dc voltage step, while measuring both the electrical and optical responses. By observing these responses and their corresponding rates of change, it is possible to infer an approximate value of the dc bias saturation voltage. This is usually the point at which the rate of change becomes negligible. In the present work, the dc bias voltage increment was set to 0.1 V and the time step to 6 minutes. The dc saturation voltage determined using this method is equal to 4.4 V. Upon extraction of the dielectric characteristics of the EC cell under test, it will be possible to establish the dielectric breakdown voltages for the activated and non-activated states.

**Figure 4:**
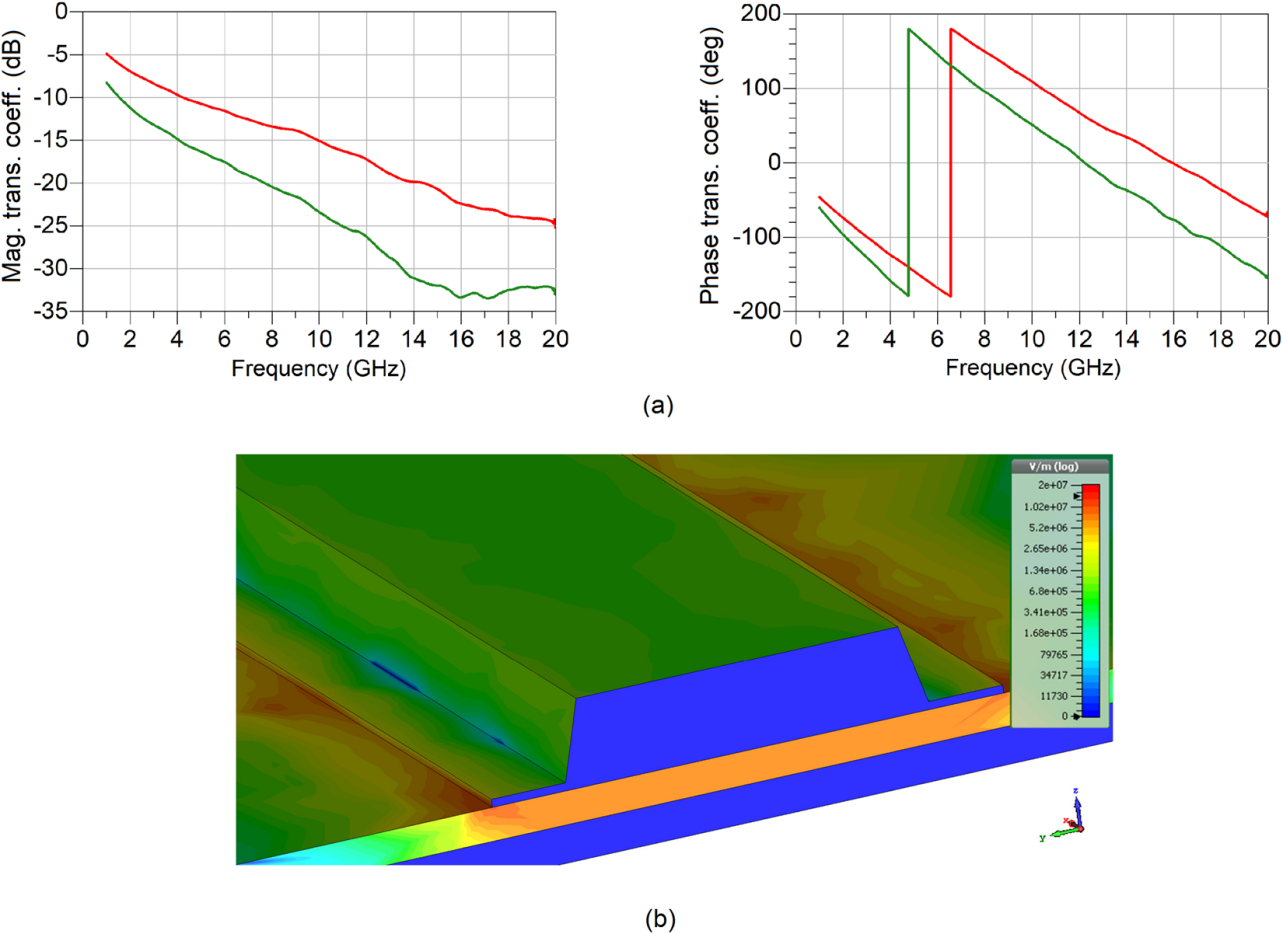
Measured electrical response of EC cell. 0 V (red) and 4.4 V (green) states (**a**), transmission coefficient, ${S}_{{21}_{E{C}_{eff}}}={e}^{-{\gamma }_{{EC}_{eff}}\ast {L}_{EC}}$, of reflection-less EC line. (**b**) Electric field distribution on EC structure at 1 GHz.

The scattering parameters of the measurement device, containing the proposed EC cell of Fig. 3 also include the effect of the CPW-microstrip transitions. This effect is taken into account using a previously published method^28^, to yield the matrix of the scattering parameters of the EC test cell i.e. without the effect of the transitions. Due to the symmetry and reciprocity of the cell, the number of unknowns in the scattering matrix is four, while the number of unknowns to be determined is two – dielectric permittivity and loss tangent. By imposing the condition that the scattering matrix needs to be reflection-less, i.e. perfectly impedance-matched, the number of unknowns in the scattering matrix reduces to two and is fully described by its complex effective propagation constant, ${\gamma }_{E{C}_{eff}}$. The measured transmission coefficient can now be represented as ${S}_{{21}_{E{C}_{eff}}}={e}^{-{\gamma }_{{EC}_{eff}}\ast {L}_{EC}}$, Fig. 4(a). The complex propagation constant contains not only the information about the EC cell stack, but is also affected by the parasitic EM propagation through the electrically thin microstrip line and the dielectric directly above it, air in this case. Viewed strictly, the dielectric parameters of the EC structure need to be represented by a second rank, in-plane-spatially dependent tensor.

1 $$(\begin{array}{c}{{\rm{D}}}_{{\rm{x}}}\\ {{\rm{D}}}_{{\rm{y}}}\\ {{\rm{D}}}_{{\rm{z}}}\end{array})=(\begin{array}{ccc}{\bar{{\rm{\varepsilon }}}}_{\parallel } & 0 & 0\\ 0 & {\bar{{\rm{\varepsilon }}}}_{\parallel } & 0\\ 0 & 0 & {\bar{{\rm{\varepsilon }}}}_{\perp }\end{array})(\begin{array}{c}{{\rm{E}}}_{{\rm{x}}}\\ {{\rm{E}}}_{{\rm{y}}}\\ {{\rm{E}}}_{{\rm{z}}}\end{array})$$

**Figure 5:**
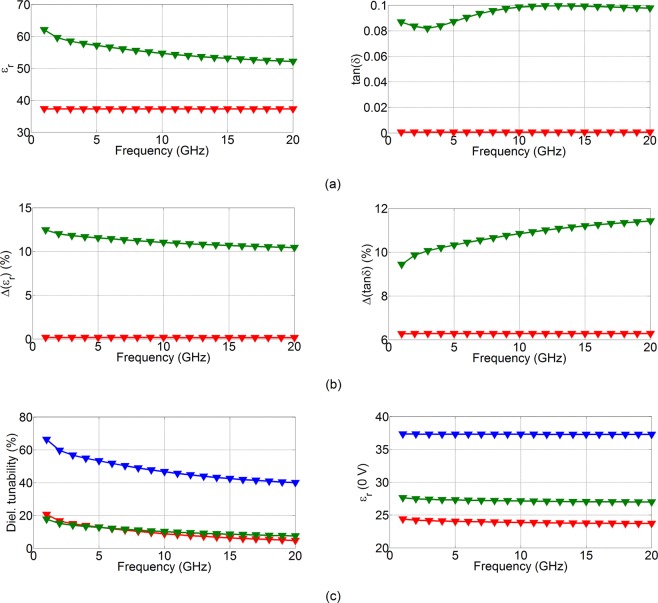
Extracted dielectric parameters of the proposed EC cell. (**a**) Extracted dielectric parameters – red unbiased 0 V, green biased 4.4 V. (**b**) Variation of observed values from 2 samples. (**c**) Left: comparison of dielectric tunabilities reported previously^20^ (red and green for cells 1 and 2, respectively) and present work (blue) and right: comparison of dielectric permittivity at 0 dc bias voltage for the previous work^20^ (red and green for cells 1 and 2 respectively) and present work (blue).

Here, ${\bar{{\rm{\varepsilon }}}}_{\parallel }$ is the z-direction dependent, complex dielectric characteristics of the individual layers WO_3_ and LiNbO_3_, while the ${\bar{{\rm{\varepsilon }}}}_{\perp }$ component represents the complex dielectric characteristics of the EC stack under investigation. Since the dominant propagation mode on the proposed structure is QTEM, there exists no field variation in the direction of propagation, x-axis. Further, since the ratio of the width of the top electrode of the EC structure of Fig. 3 to the EC structure height is very large (about 16), it can be reasonably well-assumed that the EM fields are primarily confined to the area under the top electrode, Fig. 4b, with little variation in the tangential plane. This infers that the EM propagation on the top electrode can be fully described by the knowledge of ${\bar{\varepsilon }}_{\perp }$, which in the present case can be assumed to be homogeneously distributed in the entire volume under the electrode. It may be pertinent now to refer to ${\bar{{\rm{\varepsilon }}}}_{\perp }$ as ${\bar{{\rm{\varepsilon }}}}_{{\rm{r}}}$ of the EC-stack whose dielectric characteristics are to be determined. In other words, the EC-cell stack can be described by a single, composite dielectric characteristic, where ${\bar{{\rm{\varepsilon }}}}_{\perp }={\bar{{\rm{\varepsilon }}}}_{{\rm{r}}}={{\rm{\varepsilon }}}_{{\rm{r}}}-j{{\rm{\varepsilon }}}_{{\rm{i}}}$ and $\tan (\delta )={{\rm{\varepsilon }}}_{{\rm{i}}}/{{\rm{\varepsilon }}}_{{\rm{r}}}$. However, even with these assumptions, analytical extraction of the unknown dielectric parameters of the EC cell stack from the complex propagation characteristics is impossible. This is further complicated by the fact that the top and bottom conductors are electrically thin in the frequency range from 1–20 GHz, which presents itself not only as increased losses, but it also affects the real part of the dielectric permittivity. Therefore, the extraction of the dielectric parameters is performed numerically, using a Finite Integration Technique. The extracted results are shown in Fig. 5a.

Dielectric tunability is shown to be 66% at 1 GHz and 40% at 20 GHz. The reduction in the tuning range as a function of frequency is due to the skin effect exhibited by the chromic layer (WO_3_) and can be reduced by reducing its thickness. For the purpose of repeatability and the validation of the manufacturing process, an additional EC cell with the same dimensions has also been assessed for its dielectric performance in a manner already explained. Figure 5b shows the percentage difference in the extracted dielectric parameters between the two cells, obtained using ${\rm{\Delta }}({\varepsilon }_{{\rm{r}}})=\frac{{{\rm{\varepsilon }}}_{{\rm{r}}{\rm{\max }}}-{{\rm{\varepsilon }}}_{{\rm{r}}{\rm{\min }}}}{{{\rm{\varepsilon }}}_{{\rm{r}}{\rm{\min }}}}\times 100 \%$ for the dielectric permittivity and ${\rm{\Delta }}(\tan \,\delta )=\frac{\tan \,({\delta }_{{\rm{\max }}})-\,\tan ({\delta }_{{\rm{\min }}})}{\tan \,({\delta }_{{\rm{\min }}})}\times 100 \%$ for the loss tangent. It is encouraging to see that the variation of the dielectric parameters is rather small, standing, in the worst case, at below 12% for both the dielectric permittivity and loss tangent. This points to a reasonable repeatability of the extracted results, however, with room for improvement. It is, however, interesting that the variation in the dielectric parameters in the actuated state is always higher compared to the non-actuated state. This points to an uncertainty of the exact values of the ON states. A possible reason for this lies with the incomplete Li ion intercalation into the WO_3_ chromic layer. Figure 5c presents a comparison between the dielectric tunability of the present work and the dielectric tunability of the authors’ previous work^20^. Here it is very encouraging to see that the dielectric tunability has been increased by no less than three times compared to the state-of-the-art solutions. The same figure also shows that the values of the dielectric permittivity at 0 dc bias voltage of the present work are higher than those previously reported. This is important to the design of miniaturised tuneable devices, such as phase shifters, frequency tuneable antennas and filters.

**Figure 6:**
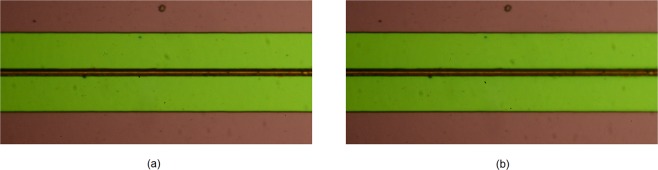
Top view micrographs taken in the z-direction with respect to device of Fig. 3 showing the measured colour change of the proposed EC cell. (**a**) Cell in the unbiased state (0 V). (**b**) Cell in the biased state (4.4 V).

**Figure 7:**
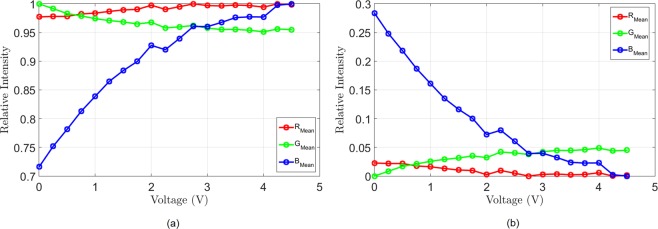
Decomposition of the optical response of the EC cell into R-G-B colour components. Index “mean” refers to the normalisation of the recorded mean intensity of a particular colour component at a prescribed dc bias voltage applied for 6 minutes. (**a**) Reflection spectrum, (**b**) absorption spectrum.

The optical response of the proposed new cell is examined next. It needs to be stated at this point that visual detection of the colouring of the chromic (WO_3_) layer is quite difficult under white light illumination, due to the fact that the chromic layer is obscured by the presence of the top electrolyte (LiNbO_3_) layer, as shown in Fig. 2. It is therefore expected that the colour efficiency of the proposed cell structure with the opaque LiNbO_3_ electrolyte is rather modest, however, the contrast between the non-actuated and actuated states can be greatly improved in the visible range by using transparent electrolytes^29^. In this way, the proposed EC structure will not only yield a high dielectric tunability, but it will fully retain its excellent colouring characteristics. Figure 6 depicts two micrographs of the proposed cell taken at 0 V and 4.5 V. Compared to the standard EC cell^20^, (Fig. S1 of the Supplementary Document), this figure indicates a rather small, however, still perceptible change in colour, not only in the vicinity of the top microstrip line, but across the entire region. In order to quantify the change in colour, the proposed cell was actuated by gradually increasing the dc bias voltage from 0 to 4.5 V, in steps of 0.25 V. The cell was kept at each dc bias step for 6 minutes and a micrograph was taken every 15 seconds, resulting in a total of 456 micrographs. These images are then decomposed into the constituent Red-Green-Blue (RGB) components and averaged across the 6 minute interval of each voltage step. A video file obtained from the images is available as a Supplementary Video File (Video 1). Each component is then normalised to the maximum value of its corresponding colour channel, Fig. 7. The decomposition of the images clearly shows that a change of colour has taken place, however, there is an important difference compared to the standard EC cells^20^, (Fig. S2 of the Supplementary Documents). In the standard EC cells^20^ the R and G components exhibit a strong change upon dc bias actuation, while the B component remains only mildly affected. In the proposed cell, the situation is reversed – the biggest change is observed in the B component, while the R and G components are less significantly affected. A probable cause is the filtering action of the top electrolyte layer.

## Discussion

The recent progress in the research on tuneable dielectrics benefitted from the discovery that alongside optical tunability, standard inorganic EC materials also exhibit voltage-bias induced dielectric tunability^19,20^. Furthermore, not only is their relative dielectric tunability tuneable, but it was also shown that the manipulation of the heights of its structural components results in the variation of its absolute levels of the dielectric permittivity^20^. In this aspect, the amount of degrees of freedom afforded by these new tuneable materials are not possible with other bulk tuneable media. Nevertheless, the extent of the dielectric tunability of traditional EC cells is of the order of about 20% and it limited by the channel shortening effect due to the dielectric-conductor transition of the chromic and ion storage layers. Our present study undoubtedly proves that the re-arrangement of the constituent layers of the standard EC cell, made with a view of avoiding the channel shortening effect results in a significant increase in the dielectric tunability, up to over 65% and no less than 40% in the frequency range from 1–20 GHz. Our results pertain to the standard chemical composition of EC cells – WO_3_ and LiNbO_3_, however, we confidently expect that a different combination of electrolytes and transition metal oxides will yield even higher levels of dielectric tunability. Thus, we truly believe that the proposed structure of the EC cell will substantially contribute to the blooming field of tuneable materials research in physical sciences.

## Supplementary information

Video 1

Electro-chromic structure with a high degree of dielectric tunability_Supplementary_file
