# Supplementary material for: Electro-chromic structure with a high degree of dielectric tunability
Source: Sci Rep. 2019 Jul 24;9:10773. doi: 10.1038/s41598-019-47233-1 (PMC6656740; doi:10.1038/s41598-019-47233-1)
Supplement: Supplementary file 2 — Electro-chromic structure with a high degree of dielectric tunability_Supplementary_file [file 41598_2019_47233_MOESM2_ESM.pdf]

Supplementary file for

Electro-chromic structure with a high degree of dielectric tunability

S. Bulja[1*], R. Kopf[2*], A. Tate, T. Hu , R. Cahill, M. Norooziarab, D. Kozlov, P. Rulikowski and W. Templ

*Corresponding authors. E-mail: senad.bulja@nokia-bell-labs.com and rose.kopf@nokia-bell-labs.com

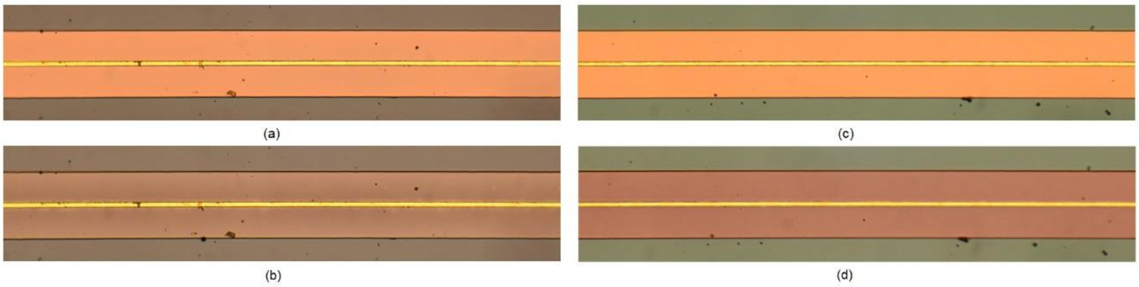

Figure S 1 *Photographs showing the measured colour change of the EC cells. (a), cell 1 in the unbiased state (0 V). (b), cell 1 in the biased state (7.1 V). (c), cell 2 in the unbiased state (0 V). (d), cell 2 in the biased state (9 V)*[20].

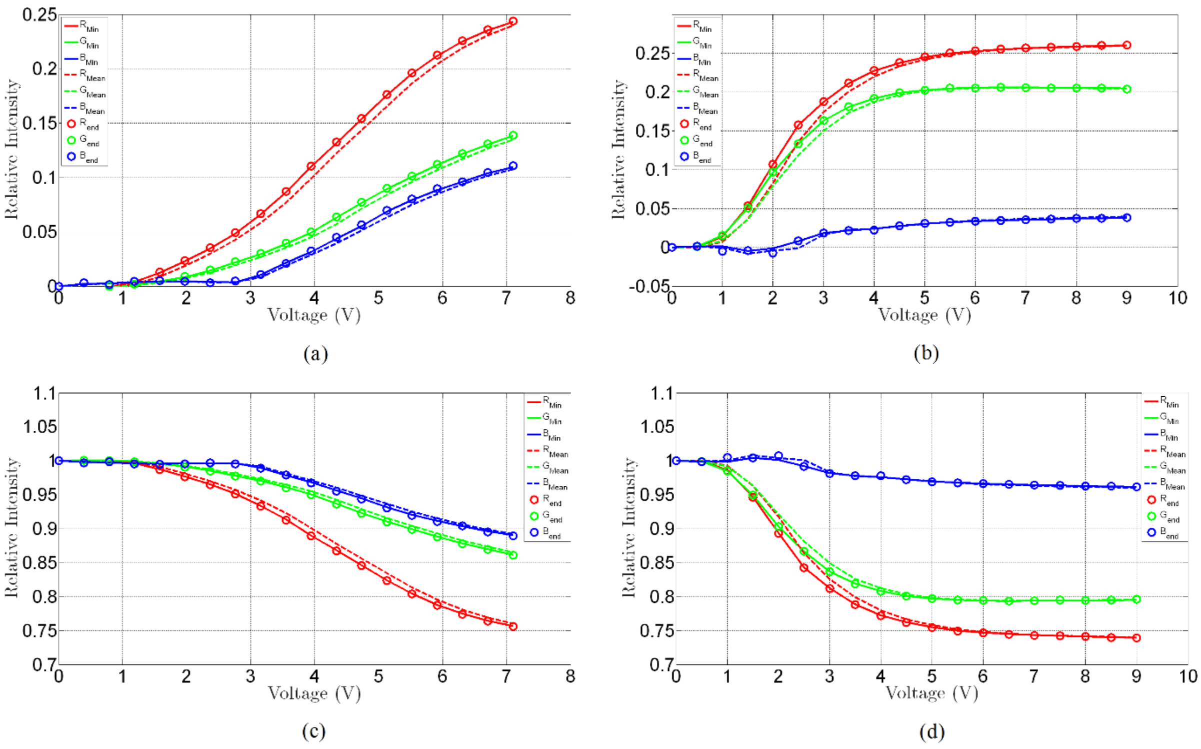

Figure *S 2* *Decomposition of the optical response of EC cells into R-G-B colour components. Index "min" refers to the normalisation of the recorded minimum intensity of a particular colour component at a prescribed dc bias voltage applied for 6 minutes, index "mean" refers to the normalisation of the recorded average of a particular colour component at a prescribed dc bias voltage applied for 6 minutes and index "end" refers to the normalisation of the recorded end value of a particular colour component at a prescribed dc bias voltage applied for 6 minutes.* ***(a),*** *cell 1, absorption spectrum,* ***(b),*** *cell 2, absorption spectrum,* ***(c),*** *cell 1 reflection spectrum and* ***(d),*** *cell 2 reflection spectrum*[20]
